# Comparisons of the Rates of Large-for-Gestational-Age Newborns between Women with Diet-Controlled Gestational Diabetes Mellitus and Those with Non-Gestational Diabetes Mellitus

**DOI:** 10.3390/clinpract14020041

**Published:** 2024-03-25

**Authors:** Sirida Pittyanont, Narongwat Suriya, Sirinart Sirilert, Theera Tongsong

**Affiliations:** 1 Department of Obstetrics and Gynecology, Prapokklao Hospital, Chanthaburi 22000, Thailand; barbieaee@hotmail.com (S.P.); teesuriya@gmail.com (N.S.); 2 Department of Obstetrics and Gynecology, Faculty of Medicine, Chiang Mai University, Chiang Mai 50200, Thailand

**Keywords:** diet control, gestational diabetes mellitus, large-for-gestational-age, pregnancy

## Abstract

(1) *Objectives*: The primary objective is to compare the rate of large-for-gestational-age (LGA) between women with diet-controlled gestational diabetes mellitus (GDM) and those with non-GDM, and to assess whether or not diet-controlled GDM is an independent factor of LGA fetuses. The secondary objectives are to compare the rates of other common adverse pregnancy outcomes, such as preeclampsia, cesarean section rate, preterm birth, and low Apgar score, between pregnancies with diet-controlled GDM and non-GDM pregnancies. (2) *Methods*: A retrospective cohort study was conducted on singleton pregnancies, diagnosed with GDM and non-GDM between 24 and 28 weeks of gestation, based on a two-step screening test. The prospective database of the obstetric department was accessed to retrieve the records meeting the inclusion criteria, and full medical records were comprehensively reviewed. The patients were categorized into two groups, GDM (study group) and non-GDM (control group). The main outcome was the rate of LGA newborns, and the secondary outcomes included pregnancy-induced hypertension, preterm birth, cesarean rate, low Apgar scores, etc. (3) *Results*: Of 1364 recruited women, 1342 met the inclusion criteria, including 1177 cases in the non-GDM group and 165 (12.3%) in the GDM group. Maternal age and pre-pregnancy BMI were significantly higher in the GDM group. The rates of LGA newborns, PIH, and cesarean section were significantly higher in the GDM group (15.1% vs. 7.1%, *p*-value < 0.001; 7.8% vs. 2.6%, *p*-value = 0.004; and 54.5% vs. 41.5%, *p*-value = 0.002; respectively). On logistic regression analysis, GDM was not significantly associated with LGA (odds ratio 1.64, 95% CI: 0.97–2.77), while BMI and gender were still significantly associated with LGA. Likewise, GDM was not significantly associated with the rate of PIH (odds ratio: 1.7, 95% CI: 0.825–3.504), while BMI and maternal age were significantly associated with PIH, after controlling confounding factors. (4) *Conclusions*: The rates of LGA newborns, PIH, and cesarean section are significantly higher in women with diet-controlled GDM than those with non-GDM. Nevertheless, the rates of LGA newborns and PIH are not directly caused by GDM but mainly caused high pre-pregnancy BMI and advanced maternal age, which are more commonly encountered among women with GDM.

## 1. Introduction

Macrosomia, defined as a birth weight ≥4000 g, and large-for-gestational-age (LGA), defined as a birth weight greater than the 90th percentile of each gestational week [1,2], are two of the most common obstetric problems because of their associations with increased maternal and perinatal morbidity/mortality, such as shoulder dystocia, postpartum hemorrhage, and birth trauma [3,4,5]. The rate of macrosomia is approximately 4–10% of term pregnancies and is currently increasing by 10–25% worldwide [6,7,8,9,10], particularly in developed countries. Gestational diabetes mellitus (GDM) is one of the most common risk factors for LGA fetuses [7,8,9,10]. Early diagnosis and treatment of GDM is helpful in the prevention of macrosomia and associated complications [2].

GDM is a carbohydrate metabolism disorder associated with insulin resistance during pregnancy, with an incidence of 7% of pregnant women [2,11]. The risk factors for GDM include advanced maternal age, a history of GDM, macrosomia, or a congenital anomaly of unknown cause in previous pregnancies. GDM is basically divided into two classes [2,12]: Class A1 or diet-controlled GDM, accounting for 86% [11], and Class A2, requiring medication treatment

Women with GDM or obesity have a higher prevalence of LGA newborns [13,14]. In a large study, the prevalence of LGA newborns in mothers without GDM was 7.7% in normal-weight women and 12.7% in obese women, and that in mothers with GDM was 13.6% in normal-weight women and 22.3% in obese women [13]. In addition to an increased risk of LGA, GDM increases the risk of gestational hypertension, preeclampsia, and caesarean section.

Currently, though the association between GDM and LGA is already known, the studies on the effectiveness of GDM control, especially diet control, in the prevention of LGA/macrosomia have been published in a very limited numbers [15,16,17,18] in spite of the fact that controlling GDM with diet is a well-accepted standard treatment for GDM. In fact, whether or not bad obstetric outcomes among GDM under good control with diet can be effectively prevented is yet to be elucidated. Importantly, most previous studies were conducted in a milieu of ideal condition under research settings, whereas studies on the effectiveness of diet control for GDM in actual practice have not been thoroughly evaluated. Therefore, we conducted this study to assess the effectiveness of diet control for GDM in the real practice of service settings after implementation of the same guideline for more than 5 years. The primary objective is to compare the incidence of LGA newborns between women with diet-controlled GDM and those with non-GDM, and to assess whether or not diet-controlled GDM is an independent factor for LGA fetuses. The secondary objectives are to compare the rate of other common adverse pregnancy outcomes, such as preeclampsia, cesarean section rate, preterm birth, and low Apgar score, between pregnancies with diet-controlled GDM and non-GDM pregnancies.

## 2. Materials and Methods

A retrospective cohort study was conducted to compare the rate of large-for-gestation-age (LGA) fetuses between pregnancies with GDM under diet control and those with non-GDM at Prapokklao Hospital, a tertiary care center, in Chanthaburi, Thailand. The study population was pregnant women attending our antenatal care clinic between 1 January 2019 and 31 December 2019. The study was ethically approved by the local Institutional Review Boards, Prapokklao Hospital Ethic Committee (Research ID COA no. 028/65, date of approval 23 March 2022). The study was based on our prospective database from the obstetric department. The database was firstly accessed to retrieve the records of women undergoing GDM screening during 24–28 weeks of gestation, and then full medical records were comprehensively reviewed by the authors to validate data. The inclusion criteria are as follows: (1) singleton pregnancy; (2) reliable gestational age, based on accurate last menstrual period consistent with fetal biometry by ultrasound examination in the first half of pregnancy, using Voluson E8 machine (GE Healthcare Ultrasound, Milwaukee, WI), equipped with transabdominal 2 to 4 MHz curvilinear transducers; (3) maternal age of greater than 18 years; (4) first visit to antenatal care in the first half of the pregnancy; and (5) low-risk pregnancy, with no underlying medical disease. The exclusion criteria are as follows: (1) taking medications that might interfere with blood glucose levels, e.g., corticosteroids, thyroid hormone, etc.; (2) known diagnosis of pre-gestational diabetes mellitus; (3) GDM that required medication or insulin to control blood glucose; (4) obstetric complications, such as hyperemesis gravidarum; (5) medical diseases, such as heart disease, kidney diseases, vascular destructive diabetes, and chronic hypertension, that are significantly associated with adverse pregnancy outcomes, such as fetal growth restriction; (6) neonates with chromosome abnormalities or congenital anomalies; and (7) loss to follow-up, unknown pregnancy outcomes, or incomplete data.

The definite diagnosis of GDM was made at a gestational age between 24 and 28 weeks, based on a two-step test recommended by the National Diabetes Data Group [2,12]. Firstly, the 50 g glucose screening test, requiring fasting, was performed; if the result was negative (the measured glucose level was lower than the cut-off value: 140 mg/dL), GDM was ruled out, and the women would be categorized as the control group or non-GDM group. In cases in which the test was positive (higher than 140 mg/dL), the 100 g, 3 h oral glucose tolerance test was performed, and this was considered to be positive if two or more plasma glucose values were higher than the thresholds (fasting glucose level > 95 mg/dL, 1 h level > 180 mg/dL, 2 h level > 155 mg/dL, and 3 h level > 140 mg/dL), and the patients were categorized as the study group or the GDM group. The women with negative tests for the 100 g, 3 h oral glucose tolerance test were also categorized as the non-GDM group.

The women in the non-GDM group were treated with the normal standard care of the antenatal clinic for low-risk pregnancies, without specific intervention. The women in GDM group were counseled by the nutritionists for diet control and were followed up to keep blood glucose levels less than the upper target levels: 95 mg/dL for fasting levels, 140 mg/dL for 1 h levels, and 120 mg/dL for 2 h levels. The dietary guideline followed the standard recommendation [2,19], including a daily amount of carbohydrates of 175 g, or ~35% (33–40%) of a daily 2000-calorie diet. The remaining calories are apportioned to give 20% as protein and 40% as fat. The patients were advised to have three small- to moderate-sized meals and one or more snacks each day, not to skip meals and snacks, and to keep the amount and types of food (carbohydrates, fats, and proteins) about the same from day to day. The recommended diet included plenty of whole fruits and vegetables, moderate amounts of lean proteins and healthy fats, moderate amounts of whole grains, such as rice, cereal, pasta, and bread, plus starchy vegetables, such as corn and peas, and fewer foods that have a lot of sugar, such as soft drinks, fruit juices, and pastries. Typically, insulin treatment was added to diet control in cases of blood glucose levels higher than the target levels, mentioned above, and these patients were excluded from analysis. All recruited pregnancies were recorded for demographic data, such as maternal age, parity, pre-pregnancy weight and height as well as body mass index (BMI: calculated by dividing weight in kg by height squared in meters), ethnicity, and obstetric history. The women were followed up for pregnancy outcomes, such as birth weight, gestational age at delivery, route of delivery, Apgar scores at one and five minutes, obstetric complications such as preeclampsia, gestational hypertension, etc.

The definitions described in this research are as follows: (1) Preterm birth is defined as delivery after 20 complete weeks and before 37 complete weeks of pregnancy. (2) Large-for-gestational-age (LGA) newborns is defined as a birth weight greater than the 90th percentile for each gestational week based on the Thai fetal growth curve. (3) Preeclampsia is defined as a new onset of maternal hypertension (blood pressure of 140/90 mmHg or greater) after 20 weeks together with a new onset of proteinuria (defined as 24 h urine protein of 300 mg or more). (4) Gestational hypertension is defined as a new onset of maternal hypertension (blood pressure of 140/90 mmHg or greater) after 20 weeks without proteinuria. (5) Pregnancy-induced hypertension (PIH) is defined as a new onset of maternal hypertension (blood pressure of 140/90 mmHg or greater) after 20 weeks, with or without proteinuria. (6) Low Apgar score is defined as a score of less than 7 at 1 and 5 min, indicating non-reassuring neonatal well-being, for which intensive care is needed.

The primary outcomes were large-for-gestational-age newborns, defined as a birth weight greater than the 90th percentile of the reference ranges, and macrosomia, defined as birth weight greater than 4000 g.

Statistical analysis: Statistical procedures were performed using the Statistical Package for the Social Sciences (SPSS) program version 26.0 (IBM Corp. Released 2019. IBM SPSS Statistics for Windows, Version 26.0, Armonk, NY, USA). The continuous variables are presented as mean  ±  SD or median (IQR) according to normality of distribution, while the categorical variables are presented as number and percentage. In comparison to the demographic data and pregnancy outcomes, categorical data were compared using a chi-square test, and the continuous data were compared using a Student’s *t*-test or Mann–Whitney U test as appropriate. Logistic regression analysis was used as a multivariate analysis to control confounding factors of large-for-date fetuses. The statistical significance was considered if a *p*-value was less than 0.05.

## 3. Results

A total of 1364 pregnant women were recruited. Of them, 1342 pregnant women met the inclusion criteria and were available for analysis, including 165 cases in the group of diet-controlled gestational diabetes mellitus (GDM) and 1177 cases in the group of non-gestational diabetes mellitus (non-GDM). The prevalence of GDM was 12.3% (165/1342). Regarding baseline characteristics of the women, maternal age and maternal BMI, as well as body weight were significantly higher in the group of GDM, whereas the others were not significantly different, as presented in Table 1. In comparisons of pregnancy outcomes, on univariate analysis, the rates of LGA newborns, PIH, and cesarean section were significantly higher in the GDM group (15.1% vs. 7.1%, *p*-value < 0.001; 7.8% vs. 2.6%, *p*-value = 0.004; and 54.5% vs. 41.5%, *p*-value = 0.002, respectively). Note that though the rate of LGA newborns was significantly higher in the GDM group, the rate of macrosomia was not significantly different between both groups. Notably, the preterm birth rate had a tendency to increase in the GDM group, but not significantly, whereas gestational age was slightly, but significantly, lower in the GDM group (*p*-value = 0.021).

Of the recruited pregnant women, the prevalence of LGA was 8.1% (109/1342). On univariate analysis, maternal age, weight, height, BMI, male fetuses, and GDM were significantly associated with an increased risk of LGA, as presented in Table 2. However, on logistic regression analysis, GDM was not significantly associated with LGA (odds ratio [OR]: 1.64, 95% confidence interval [CI]: 0.97–2.77), while BMI and gender were still significantly associated with LGA, as presented in Table 3. Likewise, on logistic regression analysis, GDM was not significantly associated with the rate of PIH (odds ratio: 1.7 (95% CI: 0.825–3.504)), while pre-pregnancy BMI and advanced maternal age were still significantly associated with PIH, as presented in Table 3.

## 4. Discussion

Insights gained from this study are as follows: (1) The prevalence of GDM, based on risk approach screening, among women in our hospital (central part of Thailand) was relatively high (12.3%), when compared with that reported in Western countries. (2) The overall prevalence of LGA infants among women with diet-controlled GDM was still higher than that in the control. (3) The pre-pregnancy BMI of the women with GDM was significantly higher than that of the control. (4) On multivariate analysis, GDM itself did not significantly increase the risk of LGA infants, while BMI was still an independent risk factor for LGA. In other words, the increase in prevalence of LGA among women with diet-controlled GDM was not influenced by GDM per se, but it was rather caused by higher pre-pregnancy BMI, which was more commonly found in women with GDM than those with non-GDM. This study indicates that proper diet control for GDM seems to be effective in the prevention of LGA. However, to be more effective in the prevention of LGA, together with GDM control, we have to focus on maintaining pre-pregnancy BMI within normal range or normal maternal weight prior to pregnancy. Note that maternal age is significantly associated with an increased risk of GDM and LGA. Nevertheless, maternal age is not an independent factor for LGA after controlling other confounding factors. (5) Likewise, pregnancy-induced hypertension (PIH), including preeclampsia and gestational hypertension, was significantly higher among diet-controlled GDM, but such an increase was influenced by underlying risk factors, including high maternal BMI and advanced maternal age instead of GDM itself. This study emphasizes the importance of diet control for GDM and provides evidence that, though it is already known that GDM increases the risk of LGA newborns, diet-controlled GDM per se does not increase the risk of LGA newborns as well as PIH.

Based on multivariate analysis, diet-controlled GDM was not significantly associated with LGA. This finding indicates that diet control can effectively prevent LGA among women with GDM, simply explained by the reduction of glucose levels resulting in a decrease in fetal fat deposition. In fact, this study supports the findings reported by Ogonowski et al. [20], who showed that the overall prevalence of macrosomia was 8.1%, and was comparable in subgroups of women with and without GDM (7.7% and 8.4%, respectively; *p* = 0.905), and those also reported by Vally et al. [21], who showed no significant difference in macrosomia between women with diet-controlled GDM and those in the non-DGM group (95% CI 0.26–1.7; *p* = 0.38). Likewise, a large population-based cohort study in China reported by Hua et al. [22] also showed the same risk factors for LGA and macrosomia. Nevertheless, the overall prevalence of LGA was still higher among the GDM group, mostly associated with pre-pregnancy overweight/obesity, which increased the risk of macrosomia and LGA births independently [23,24,25,26] and was partly mediated by GDM [27].

Certainly, GDM is an independent risk factor for LGA infants [28,29,30,31], directly dosage-dependent on blood glucose levels [29,32,33]. In GDM, maternal hyperglycemia causes fetal hyperglycemia via placental transportation of glucose, leading to fetal hyperinsulinemia. As a consequence, an insulin-induced hypermetabolic state promotes excessive somatic growth. Except for the brain, most fetal organs are affected by the macrosomia that characterizes the fetus of diabetic mothers, resulting in truncal obesity and LGA fetuses [34]. Infants are anthropometrically different from other LGA neonates [35,36]. Specifically, those whose mothers are diabetic have excessive fat deposition on the shoulders and trunk, which predisposes them to shoulder dystocia or feto-pelvic disproportion. There is a direct correlation in diabetic mothers between the degree of fetal truncal asymmetry and the prevalence as well as severity of shoulder dystocia [34]. Theoretically, well-controlled GDM, as indicated by the achievement of the target glucose level, mentioned earlier, can be expected to be effective in preventing LGA [29]. Whereas glycemic control in most studies included both diet and medication and being conducted under research setting, our study specifically documented that diet-controlled GDM that is managed well without medication can also prevent LGA in the actual practice of service settings.

GDM also increases the risk of PIH [37,38], though such an increase is not as high as that seen in pre-gestational diabetes mellitus. This study demonstrated that diet control of GDM could also prevent the development of PIH. Though the overall prevalence of PIH on univariate analysis was significantly higher in the GDM group, it was not significantly different on multivariate analysis. Such an increase was associated with other risk factors, including pre-pregnancy BMI and maternal age, but was not directly influenced by GDM. As already known, high BMI and advanced maternal age are significantly associated with an increased risk of PIH [39]. In this study, BMI and maternal age were confounding factors for the development of PIH in the GDM group, which could not be prevented by glucose control. Acetylsalicylic acid (ASA) prophylaxis [40], rather than glucose control, may be expected to reduce the prevalence of PIH associated with advanced maternal age or pre-pregnancy BMI.

Notably, the cesarean section rate was rather high in our center (43.1%), and the rate was significantly higher in the GDM group. Presumably, the higher cesarean section rate might be explained by the greater prevalence of LGA fetuses in the GDM group, which consisted of the greater number of cases with high pre-pregnancy BMI. Other adverse obstetric outcomes between the two groups were comparable. Note that gestational age among women in the study group was significantly lower than that in the control group. Nevertheless, the statistically significant difference of only 0.29 weeks is unlikely to be clinically significant. Additionally, the rate of preterm birth was not significantly different between both groups. Likewise, a statistically significant difference in estimated blood loss of 38 mL is unlikely to have clinical significance.

This study points out that a strategy to reduce the prevalence of LGA as well as PIH and CSR among GDM must include controlling other risk factors as well, especially pre-pregnancy BMI. Controlling GDM alone cannot be perfectly successful in avoiding LGA.

Strengths and weaknesses: The strengths of this study are as follows: (1) The measure of diet control in the GDM group is highly reliable because of close monitoring and assessment by our nutritionist, and the retrieved data were based on a comprehensive review of the full medical records. (2) The sample size is large enough to address the primary objective of the study. (3) The results can reflect more the effectiveness of diet control for GDM in actual practice because of being derived from real practice in a service setting, where the adherence to diet control might not be perfect, rather than ideal conditions of a research setting. The weaknesses of this study are as follows: (1) The extent of the study did not cover the cases of GDM controlled with insulin. (2) The sample size might be too small for secondary outcomes with relatively low prevalence, such as low Apgar scores, perinatal mortality, and macrosomia (birth weight greater than 4000 g), which is a stronger predictor of bad obstetric outcomes than LGA newborns. (3) The data of neonatal outcomes, in particular neonatal blood glucose levels, were not evaluated.

## 5. Conclusions

This study suggests that the overall prevalence of LGA, as well as PIH, among women with GDM under good diet control is still significantly higher than that in the control group but that such an increase is associated with maternal pre-pregnancy BMI and maternal age, and not significantly associated with GDM itself. The finding implies that good diet control in women with GDM is effective in the prevention of LGA fetuses, as well as PIH. However, to reduce the overall prevalence of LGA and PIH among women with diet-controlled GDM, keeping pre-pregnancy BMI within normal limits should strongly be taken into consideration. The results of this study may be used as a reference for evaluation in other studies based on service settings and as a guideline for hospital management of GDM, especially focusing on modification of lifestyle or pre-pregnancy BMI.

## Figures and Tables

**Table 1:** Baseline characteristics and pregnancy outcomes of the women with GDM and non-GDM. GDM: Gestational diabetes mellitus.

	Non GDM (*n*: 1177)	GDM (*n*: 165)	*p*-Value
*Baseline characteristics*			
Maternal age	26.78 ± 6.22	32.59 ± 6.11	<0.001
Body weight (kg)	68.52 ± 12.28	73.7 ± 13.80	<0.001
Height (cm)	158.40 ± 6.00	157.92 ± 6.30	0.338
BMI (kg/m^2^)	27.30 ± 4.63	29.51 ± 4.97	<0.001
Parity			0.080
Primiparity	469/1177 (39.8%)	48/165 (29.1%)	
Multiparity	708/1177 (60.2%)	117/165 (70.9%)	
*Pregnancy outcomes*			
Gestational age (week)	38.66 ± 1.47	38.37 ± 1.54	0.021
Birth weight (g)	3013.47 ± 429.93	3045.64 ± 462.33	0.373
Placental weight (g)	610 ± 124	617 ± 124	0.471
Estimated blood loss (mL)	333.97 ± 6.22	371.21 ± 162.97	0.008
Preterm birth	77/1177 (6.5%)	15/165 (9.1%)	0.225
Hypertensive disorders			0.004
Gestational hypertension	10/1177 (0.8%)	6/165 (3.6%)	
Preeclampsia	21/1177 (1.8%)	7/165 (4.2%)	
Cesarean delivery	489/1177 (41.5%)	90/165 (54.5%)	0.002
Low Apgar score at 1 min	50/1177 (4.2%)	7/165 (4.2%)	0.997
Low Apgar score at 5 min	8/1177 (0.7%)	2/165 (1.2%)	0.456
Infant’s gender			0.827
Male	617/1177 (52.4%)	85/165 (51.5%)	
Female	560/1177 (47.6%)	80/165 (48.5%)	
Large-for-gestational-age	84/1177 (7.1%)	25/165 (15.2%)	<0.001
Macrosomia	40/1177 (3.4%)	9/165 (5.5%)	0.187

**Table 2:** Risk factor associated with large-for-gestational-age (LGA).

	No LGA (*n*: 1233)	LGA (*n*: 109)	*p*-Value
Maternal age	27.33 ± 6.49	29.35 ± 6.15	0.002
Body weight (kg)	68.27 ± 12.06	79.15 ± 14.01	<0.001
Height (cm)	158.16 ± 5.99	160.36 ± 6.19	<0.001
BMI (kg/m^2^)	27.29 ± 4.57	30.79 ± 5.30	<0.001
Gestational age (week)	38.59 ± 1.49	38.92 ± 1.38	0.300
Parity			0.080
Primiparity	486/1233 (39.4%)	31/109 (28.4%)	
Multiparity	747/1233 (60.6%)	78/109 (71.6%)	
Preterm	87/1233 (7.1%)	5/109 (4.6%)	0.328
Gestational diabetes	140/1233 (11.4%)	25/109 (22.9%)	<0.001
Estimated blood loss (mL)	333.07 ± 164.63	400.45 ± 195.02	0.010
Placental weight	599 ± 118	748 ± 112	<0.001
Infant’s gender			0.001
Male	629/1233 (51.0%)	73/109 (67.0%)	
Female	604/1233 (49.0%)	36/109 (33.0%)	
Birth weight	2950.80 ± 380.7	3771.06 ± 246.68	<0.001

**Table 3:** Logistic regression for large-for-gestational-age and pregnancy-induced hypertension.

	Coefficeint (beta)	*p*-Value	Odds Ratio	95% CI for Odds Ratio	
				Lower	Upper
*For large-for-gestational-age*					
Maternal age	0.0178	0.323	1.02	0.98	1.05
Parity (Multiparity)	0.2473	0.312	1.28	0.79	2.07
Body mass index (kg/m^2^)	0.1211	<0.001	1.13	1.09	1.17
Gender (Male)	0.6138	0.005	1.85	1.21	2.83
Gestational diabetes	0.4948	0.064	1.64	0.97	2.77
*For pregnancy-induced hypertension*					
Maternal age	0.094	<0.001	1.099	1.043	1.158
Parity (Multiparity)	−1.005	0.004	0.366	0.186	0.720
Body mass index (kg/m^2^)	0.131	<0.001	1.140	1.079	1.205
Infant’s gender (Male)	0.055	0.863	1.057	0.566	1.974
Gestational diabetes	0.531	0.150	1.700	0.825	3.504

## Data Availability

The raw data supporting the conclusions of this article will be made available by the authors on request.
